# Influences of Ingredients and Bakers on the Bacteria and Fungi in Sourdough Starters and Bread

**DOI:** 10.1128/mSphere.00950-19

**Published:** 2020-01-15

**Authors:** Aspen T. Reese, Anne A. Madden, Marie Joossens, Guylaine Lacaze, Robert R. Dunn

**Affiliations:** a Society of Fellows, Harvard University, Cambridge, Massachusetts, USA; b Department of Applied Ecology, North Carolina State University, Raleigh, North Carolina, USA; c Laboratory of Molecular Bacteriology, Rega Institute, Department of Microbiology and Immunology, KU Leuven, Leuven, Belgium; d VIB-KU Leuven Center for Microbiology, Leuven, Belgium; e Laboratory of Microbiology, Department of Biochemistry and Microbiology, Faculty of Science, Ghent University, Ghent, Belgium; f Puratos Center for Bread Flavour, Puratos Corporation, Vith, Belgium; g Center for Macroecology, Evolution, and Climate, Natural History Museum of Denmark, University of Copenhagen, Copenhagen, Denmark; h German Centre for Integrative Biodiversity Research (iDiv), Leipzig, Germany; University of Wisconsin—Madison

**Keywords:** *Lactobacillus*, *Saccharomyces*, skin microbiome, sourdough

## Abstract

Sourdough starters are naturally occurring microbial communities in which the environment, ingredients, and bakers are potential sources of microorganisms. The relative importance of these pools remains unknown. Here, bakers from two continents used a standardized recipe and ingredients to make starters that were then baked into breads. We characterized the fungi and bacteria associated with the starters, bakers’ hands, and ingredients using 16S and internal transcribed spacer (ITS) rRNA gene amplicon sequencing and then measured dough acidity and bread flavor. Starter communities were much less uniform than expected, and this variation manifested in the flavor of the bread. Starter communities were most similar to those found in flour but shared some species with the bakers’ skin. While humans likely contribute microorganisms to the starters, the reverse also appears to be true. This bidirectional exchange of microorganisms between starters and bakers highlights the importance of microbial diversity on bodies and in our environments as it relates to foods.

**IMPORTANCE** Sourdough starters are complex communities of yeast and bacteria which confer characteristic flavor and texture to sourdough bread. The microbes present in starters can be sourced from ingredients or the baking environment and are typically consistent over time. Herein, we show that even when the recipe and ingredients for starter and bread are identical, different bakers around the globe produce highly diverse starters which then alter bread acidity and flavor. Much of the starter microbial community comes from bread flour, but the diversity is also associated with differences in the microbial community on the hands of bakers. These results indicate that bakers may be a source for yeast and bacteria in their breads and/or that bakers’ jobs are reflected in their skin microbiome.

## Abstract (precis)

Sourdough starters are complex communities of yeast and bacteria which confer characteristic flavor and texture to sourdough bread. The microbes present in starters can be sourced from ingredients or the baking environment and are typically consistent over time. Herein, we show that even when the recipe and ingredients for starter and bread are identical, different bakers around the globe produce highly diverse starters which then alter bread acidity and flavor. Much of the starter microbial community comes from bread flour, but the diversity is also associated with differences in the microbial community on the hands of bakers. These results indicate that bakers may be a source for yeast and bacteria in their breads and/or that bakers’ jobs are reflected in their skin microbiome.

## INTRODUCTION

The bacterial and fungal species present in sourdough bread have the potential to influence the flavors, aromas, shelf stability, and even nutritional quality of bread (1–3). These microorganisms vary among starters (4, 5), regions, and baking environments (6) and may even vary within starters through time (7). Recent research has begun to identify the potential for microorganisms from many sources to colonize starters, including those from flour (8, 9), the air in bakeries (10), and even, some have speculated (10), the bakers themselves. Here, we use an experiment in which we brought together bakers from around the world to begin to disentangle these influences, with a focus on the potential contribution of microorganisms from bakers’ hands and bodies.

Ultimately, all starters begin with a set of relatively simple ingredients. Often, just flour and water are mixed together and allowed to begin to ferment. Following fermentation, starters are typically composed of both yeast and acid-producing bacteria. Canonically, one or several *Lactobacillus* bacteria species produce the acid in the starter, and the yeasts produce carbon dioxide. Recent research has, however, identified a great deal more variation among starters than previously expected, including starters reliant on bacteria and yeasts other than *Lactobacillus* and *Saccharomyces* (6, 7, 11, 12).

When starters are made, the first microorganisms with the opportunity to colonize the starter are those in the flour itself. Flour has the potential to contain both species of microorganisms that lived inside the grain (endophytes) and those species found living on the outside of the grain or that colonize the grain during processing and storage (9). In theory, microorganisms in water could also colonize, but none of the common microorganisms in water systems are thought to play a major role in starters (13). The dust in the air mixed into the starter may also influence the composition of starters. Most of the bacterial genera commonly found in starters are often found in air and dust samples (14). The presence of yeasts in dust samples is, however, rare (e.g., 14). The microorganisms in air have the potential to add great variation among starters in as much as the microbiology of dust and air is strongly influenced by geography, vegetation type, and other regional factors (14).

A final potential contributor to sourdough starters is the biology of the person who makes the starter in the first place. This is particularly true for the bacteria present in starters—many of which are either human-associated species or closely related to human-associated species. Bacteria of the genus *Lactobacillus*, for example, are dominant members of human vaginal communities (15), gut communities (16), and to a lesser extent, the hands (17). For example, in one study, 2% of the bacteria on the hands of men and 6% of those on the hands of women were from the *Lactobacillaceae* family (17).

Here, we carried out an experiment to examine the correlations between the microbes present in potential sources and the microorganisms found in starters. We then separately tested the effect of these microbial communities on resultant bread traits. Eighteen bakers from fourteen countries were sent flour with which to make starter and a recipe to use in making the starter (see Fig. S1 in the supplemental material). Bakers then brought their starters with them to Sankt Vith, Belgium, where the bacteria and fungi of the flour, the starters, and the hands of the bakers were sampled. We analyzed the similarity of the bacterial and fungal communities in the starters to those in the flour and on the bakers’ hands using amplicon sequencing of ribosomal DNA on an Illumina MiSeq platform. We particularly focused on the functionally important yeasts and lactic acid bacteria. We compared the microbial community (alpha- and beta-diversity) between starters and hands, assessed the likelihood of flour, hands, and dust serving as sources for the starter community, and tested for a relationship between microbial community composition and starter acidity as well as final bread flavor.

10.1128/mSphere.00950-19.1 Fig. S1 Protocol given to bakers to produce a sourdough starter. Download FIG S1, TIF file, 1.0 MB. Copyright © 2020 Reese et al. 2020 Reese et al. This content is distributed under the terms of the Creative Commons Attribution 4.0 International license.

## RESULTS

**FIG 1:**
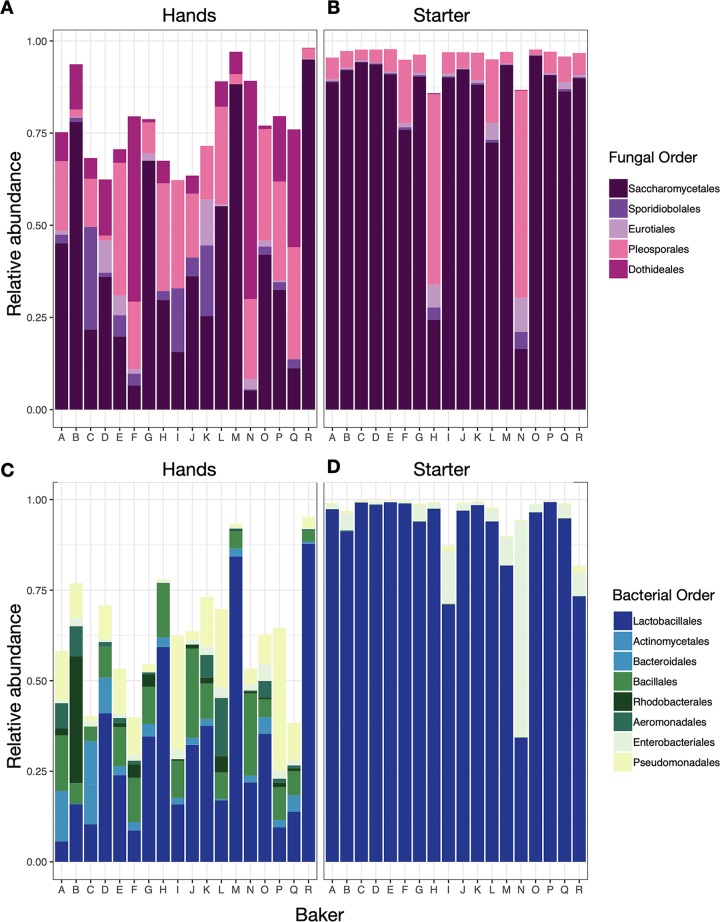
Order-level composition of the bacterial and fungal communities for hands and starters by baker identity (bakers A to R). Only orders with an average abundance of greater than 1% are shown.

**FIG 2:**
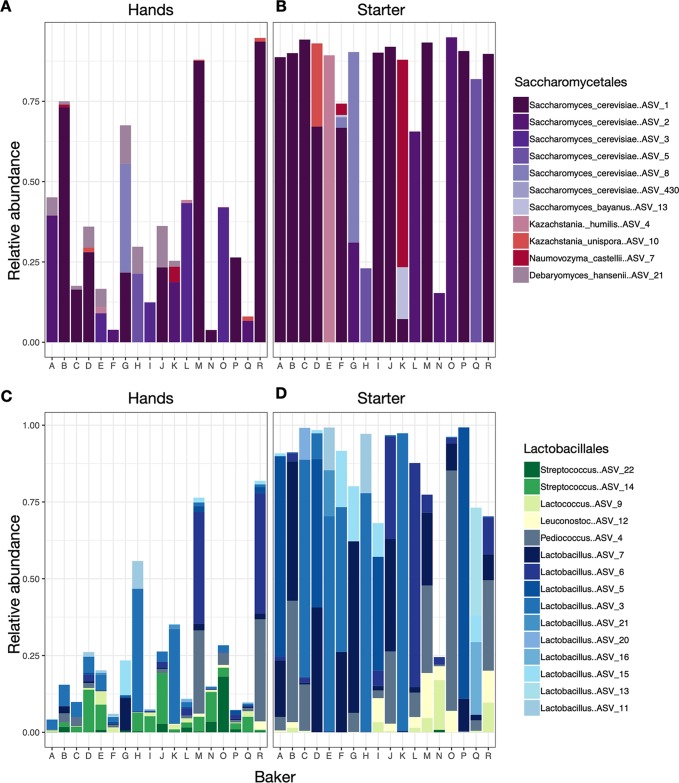
ASV-level composition of members of the *Saccharomycetales* (yeasts) and *Lactobacillales* (lactic acid bacteria) for hands and sourdough starters by baker identity (bakers A to R). Only ASVs with an average abundance greater than 1% are shown.

Regardless of identity or geography, all bakers successfully made a starter which would later be used to bake bread. Collectively, the starters were dominated by carbon dioxide-producing yeasts (order: *Saccharomycetales*) and lactic acid bacteria (order: *Lactobacillales*; Fig. 1). Baker’s hands also had high relative abundances of lactic acid bacteria and yeasts, including species which are not typically found on human skin (Fig. 1). Within these functionally important groups, we noted amplicon sequence variant (ASV)-level variation among bakers, both on their hands and in their starters (Fig. 2).

**FIG 3:**
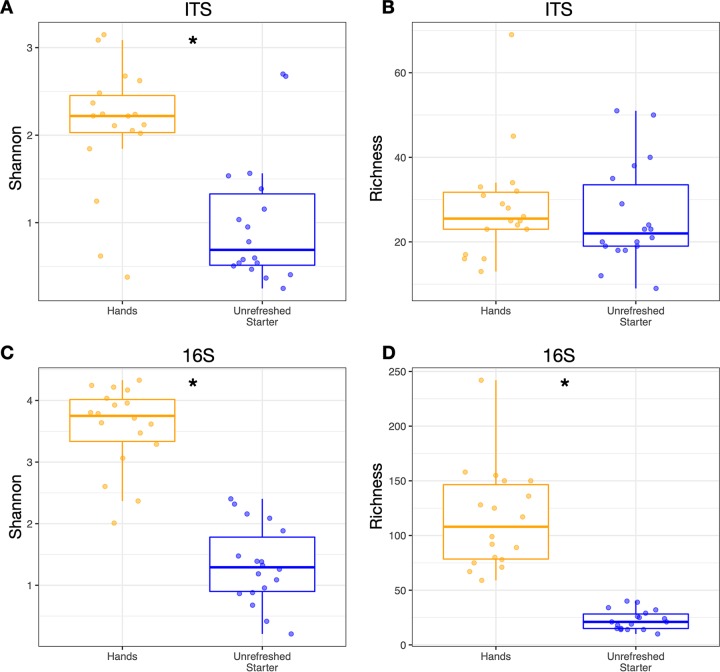
Alpha-diversity of rarefied bacterial and fungal communities differed between hands and starters. Boxplot shows the median and the first and third quartiles, and whiskers show 1.5 times the interquartile range. *, *P* < 0.05 Mann-Whitney U test.

Alpha-diversity metrics—both Shannon index and ASV richness—of rarefied bacterial and fungal communities differed between hands and starters. Compared to those of hands, the fungal communities in starters were not less diverse (*P* = 0.45, Mann-Whitney U test; Fig. 3A) but were dominated, in terms of relative abundance, by fewer taxa (*P* < 0.001, Mann-Whitney U test; Fig. 3B). Bacteria had both lower Shannon diversity (*P* < 0.001, Mann-Whitney U test; Fig. 3C) and lower richness (*P* < 0.001, Mann-Whitney U test; Fig. 3D) in starters than on hands. The differences among bakers in terms of the alpha-diversity of fungi and bacteria on their hands and in their starters were large. However, bakers with more diverse hand communities did not have significantly more diverse microbial communities in their starters (*P* > 0.4, Kruskal-Wallis test).

**FIG 4:**
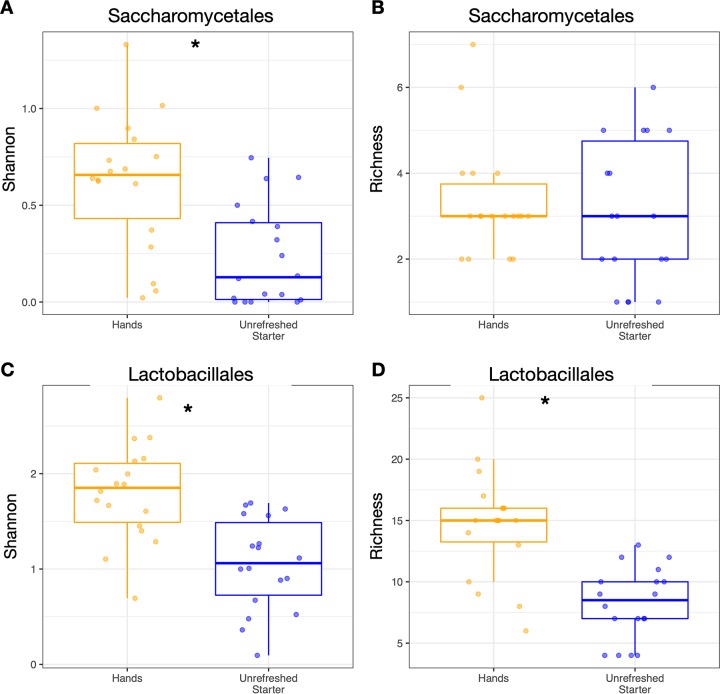
Alpha-diversity of rarefied *Saccharomycetales* (yeasts) and *Lactobacillales* (lactic acid bacteria) differed significantly between hands and starters. Boxplot shows the median and the first and third quartiles, and whiskers show 1.5 times the interquartile range. *, *P* < 0.05 Mann-Whitney U test.

Similarly, rarefied *Saccharomycetales* yeast Shannon diversity was higher on hands than in starters (*P* = 0.001, Mann-Whitney U test; Fig. 4A), although richness did not differ significantly between the two habitats (*P* = 0.54; Fig. 4B). Both measures of the diversity of *Lactobacillales* bacteria—Shannon index and richness—were higher on hands than in starters (*P* = 0.001, Mann-Whitney U test; Fig. 4C and D). Consistent with our results when considering all taxa, the diversity of either functional group on a baker’s hands was not predictive of its diversity in that baker’s starter (*P* > 0.2, Kruskal-Wallis test).

**FIG 5:**
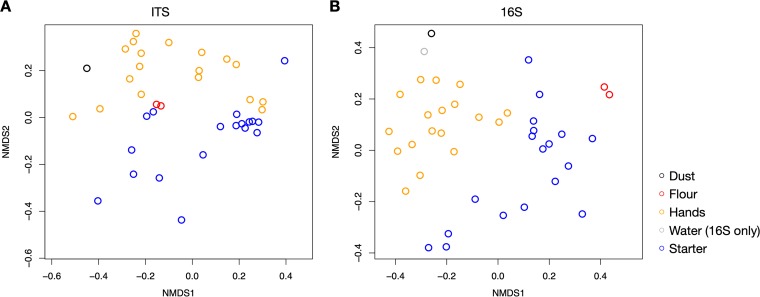
Nonmetric multidimensional scaling of fungal (A) and bacterial (B) community portions by sample type. There is clustering of sample types and, specifically, of flour samples with starters. Stress for fungal NMDS is 0.1799806 and for bacterial NMDS is 0.2079617.

Overall community composition at the ASV level differed between starters and hands (fungi, *P* = 0.001 and *R*^2^ for sample type = 0.16; bacteria, *P* = 0.001 and *R*^2^ for sample type = 0.22; permutational multivariate analysis of variance [PERMANOVA]) (Fig. 5, Fig. S3). Bray-Curtis dissimilarity was lower (i.e., communities were more similar) between starters and flour samples than between starters and hands and between starters and dust or water samples from the baking facility for both bacterial and fungal communities. In other words, starter bacterial and fungal communities were most similar to flour samples.

10.1128/mSphere.00950-19.3 FIG S3 Nonmetric multidimensional scaling of fungal (A) and bacterial (B) community portions by sample type showing starters and hands identified by baker. Stress for fungal NMDS is 0.1625475 and for bacterial NMDS is 0.1742602. Download FIG S3, TIF file, 0.3 MB. Copyright © 2020 Reese et al. 2020 Reese et al. This content is distributed under the terms of the Creative Commons Attribution 4.0 International license.

We found that there was substantial overlap both between microbial taxa present in starters and hands and between starter communities and flour samples (Table 1, Table S3). Overall, 59% of ASVs present in flour were found in at least one starter, making up 23% of all ASVs in starters. A smaller proportion of the ASVs from hands (11%) were found in starters, but these ASVs represented 46% of the ASVs present in starters. These shared ASVs represented almost all of the reads in starters (97 ± 4% of bacterial reads; 97 ± 4% of fungal reads) but fewer of the reads for hands (42 ± 21% of bacterial reads; 55 ± of 20% fungal reads). The ASVs shared only by hands and starters (i.e., those that were never found in flour) represented fewer reads in starters (58 ± 31% of bacterial reads; 32 ± of 38% fungal reads) and on hands (29 ± 12% of bacterial reads; 13 ± 15% of fungal reads). Bacterial and fungal ASVs from flour and hands were found at similarly high frequencies in starters (Table S4). Finally, hands had many more unique taxa in total (1,296) and as a proportion of all ASVs (88%) than starters or flour samples (Table 1, S3).

**TABLE 1:** All bacterial and fungal ASVs that are shared by sources^*a*^

Source	Flour	Hand	Starter
Flour	44		
Hand	74	1,296	
Starter	79	157	186
Total ASVs	133	1,463	358

a Values on the diagonal are unique to that sample type. 64 ASVs were found at least once in each of the three sample types.

10.1128/mSphere.00950-19.7 TABLE S2 List of bacterial and fungal ASVs that are shared or unique to each sample type (i.e., what is counted in Table 1). Download Table S2, XLSX file, 0.1 MB. Copyright © 2020 Reese et al. 2020 Reese et al. This content is distributed under the terms of the Creative Commons Attribution 4.0 International license.

10.1128/mSphere.00950-19.8 TABLE S3 Proportion of the shared bacterial and fungal ASVs that are exclusively shared by each source pair. Proportions on the diagonal are those unique to that sample type. Proportions are calculated with the total number of ASVs in the column header as the denominator. Download Table S3, XLSX file, 0.01 MB. Copyright © 2020 Reese et al. 2020 Reese et al. This content is distributed under the terms of the Creative Commons Attribution 4.0 International license.

10.1128/mSphere.00950-19.9 TABLE S4 Fungal (A) and bacterial (B) ASVs that are shared by each source pair. Percentages are unique shared (i.e., only in the two being compared). 40 fungal ASVs and 24 bacterial ASVs were found at least once in each of the three sample types. Download Table S4, XLSX file, 0.01 MB. Copyright © 2020 Reese et al. 2020 Reese et al. This content is distributed under the terms of the Creative Commons Attribution 4.0 International license.

We also used a Bayesian approach, SourceTracker (18), to estimate the proportion of microbes from dust, water, hands, and unknown sources that were found in sourdough starters. SourceTracker analyses indicated that starters had the highest representation of microbes found on hands (0.75 ± 0.27; Fig. S4A), followed by flour (0.07 ± 0.08) and unknown (0.14 ± 0.23). All but two starters had a proportion of at least 0.5 identified as sourced from hands. In contrast, when we trained a SourceTracker model with starters identified as the potential source of bacteria and fungi and hands as the habitat being colonized from that source (sink), we found that starters (0.25 ± 0.29), unknown (0.59 ± 0.22), and dust (0.08 ± 0.09) were all common sources (Fig. S4B).

10.1128/mSphere.00950-19.4 FIG S4 Source environment proportions for each starter (A) or baker’s hand (B) estimated using SourceTracker. Shown are estimates of proportions drawn from each environmental source. Download FIG S4, TIF file, 0.5 MB. Copyright © 2020 Reese et al. 2020 Reese et al. This content is distributed under the terms of the Creative Commons Attribution 4.0 International license.

**FIG 6:**
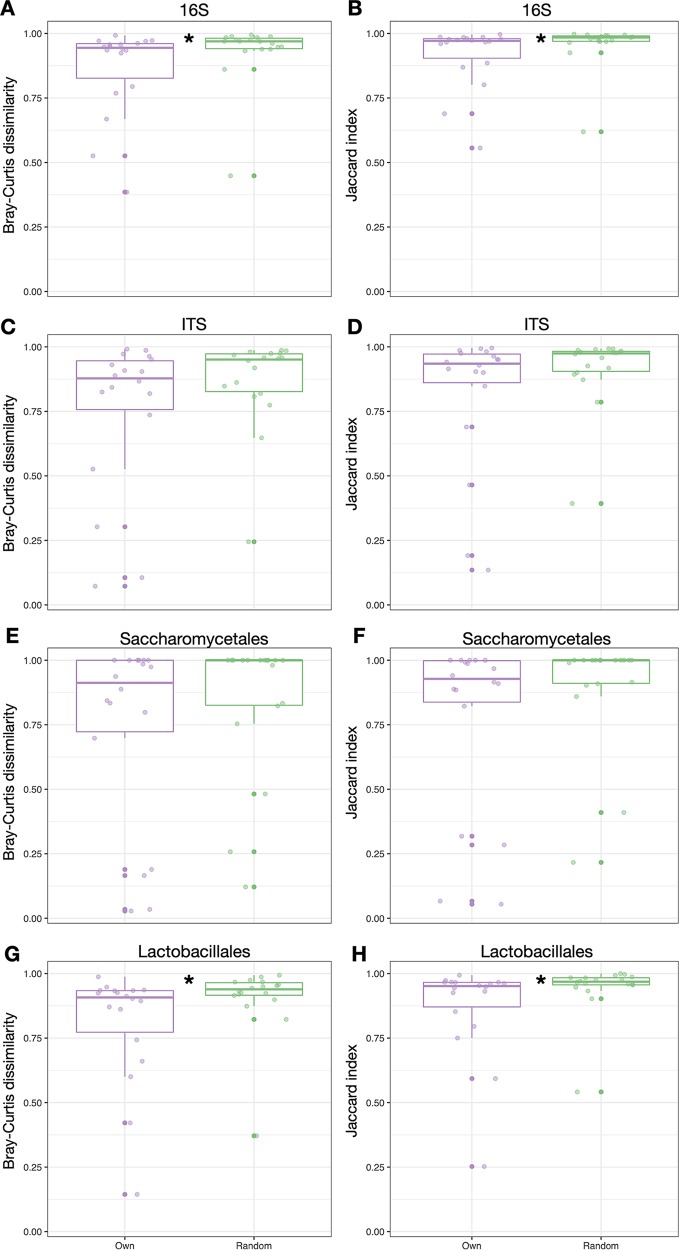
Dissimilarity in the microorganism community between a baker’s hands and that baker’s own starter tends to be lower than to a random baker’s starter. The communities of bacteria (A, B), fungi (C, D), *Saccharomycetales* (yeasts; E, F), and *Lactobacillales* (lactic acid bacteria; G, H) are compared. Boxplot shows the median and the first and third quartiles, and whiskers show 1.5 times the interquartile range. *, *P* < 0.05 Mann-Whitney U test.

Although starter communities shared many similarities with the skin microbiome of bakers’ hands, the effect of baker identity on starter composition was not strongly visible in ASV-level beta-diversity. We did not find a relationship between baker identity and beta-diversity overall (*P* > 0.05, PERMANOVA). We next tested whether the skin microbiome of the bakers’ hands was more similar to their own starter than to someone else’s using two beta-diversity metrics (Bray-Curtis dissimilarity includes abundance data; the Jaccard index is only presence-absence). We saw that the bacterial skin microbiome of bakers’ hands more closely resembled their own starter than a random starter for both Bray-Curtis dissimilarity (*P* = 0.054) and Jaccard index (*P* = 0.054, Mann-Whitney U test; Fig. 6A and B). Furthermore, bakers shared a more similar *Lactobacillales* community with their own starter compared to a random starter (Bray-Curtis, *P* = 0.028; Jaccard, *P* = 0.026; Mann-Whitney U test; Fig. 6G and H). When comparing fungal and yeast communities alone, there was a similar trend of greater similarity to one’s own starter but no significant difference (*P* > 0.1, Mann-Whitney U test; Fig. 6C to F).

**FIG 7:**
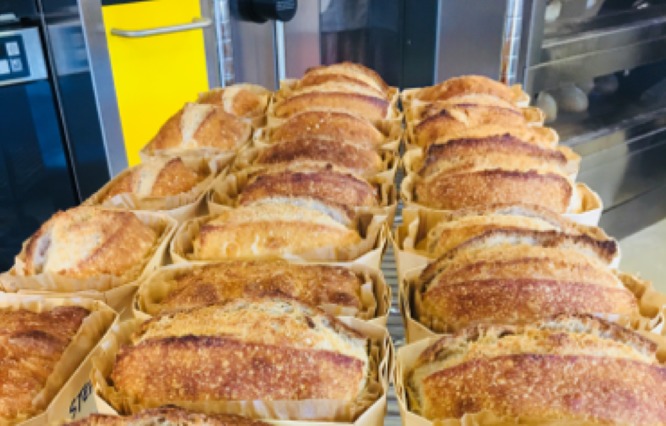
A selection of the breads baked with the sourdough starters.

As expected, the starters were acidic (pH mean, 4.50 ± 0.38; total titratable acidity, 9.68 ± 1.51) (Table S1), but the level of acidity varied among starters. The more similar any two starters were in their pH, the more similar they were in their composition (including both fungi and bacteria; *P* = 0.019, *R*^2^ for acidity = 0.16, PERMANOVA). We also assessed whether variation in the microbial community composition of starters was associated with differences in the flavor of the final bread (Fig. 7). Starters were used to bake sourdough bread loaves following a standardized recipe and protocol (Fig. S2). The resulting breads were scored for five taste characteristics (acid, cereal, creamy, fermented, fruity; Table S5). Altogether, there was no association between collective variation in these flavor characteristics and variation in microbial community composition (*P* = 0.155, Mantel test). However, variation in starter community composition was associated with variation in the “acid” taste characteristic (*P* = 0.040, *R*^2^ = 0.13, PERMANOVA). There was also an association between *Lactobacillales* composition and overall flavor (*P* = 0.025, Mantel statistic = 0.20) as well as with “acid” taste (*P* = 0.004, *R*^2^ = 0.21). In contrast, *Saccharomycetales* community composition was not related to aggregate flavor or to any individual flavor characteristic.

10.1128/mSphere.00950-19.2 FIG S2 The recipe and methods used for baking breads with the sourdough starters. Download FIG S2, TIF file, 0.2 MB. Copyright © 2020 Reese et al. 2020 Reese et al. This content is distributed under the terms of the Creative Commons Attribution 4.0 International license.

10.1128/mSphere.00950-19.6 TABLE S1 pH and TTA for each sourdough starter by baker. Starter pH and TTA were highly correlated. Download Table S1, XLSX file, 0.01 MB. Copyright © 2020 Reese et al. 2020 Reese et al. This content is distributed under the terms of the Creative Commons Attribution 4.0 International license.

10.1128/mSphere.00950-19.10 TABLE S5 Bread flavor characteristics. Breads were scored for 5 taste characteristics by a panel of judges. Average ± standard deviation and range are reported. Download Table S5, XLSX file, 0.01 MB. Copyright © 2020 Reese et al. 2020 Reese et al. This content is distributed under the terms of the Creative Commons Attribution 4.0 International license.

## DISCUSSION

Here, we show that starters generally resemble the flour used to make them. But, interestingly, a large proportion of the bacteria and yeasts in sourdough starters can also be found on the hands of bakers. Even when the recipe and ingredients for a starter and bread are identical, the diverse communities of starter bacteria and fungi that arose—due to some mix of what was on bakers’ hands, what was in the air in their kitchen, and other unknown sources—influenced the flavor of the bread.

In our study, 18 bakers used the same flour to make a single starter each and then, using the same ingredients, a single loaf of bread each. The yeasts and bacteria in starters can differ due to differences in flour type and condition (19, 20) and the additional ingredients made using a starter (21). We sought to minimize the influence of these factors, focusing instead on how differences in baker identity and location may impact otherwise standardized starters. Nonetheless, the diversity of species of bacteria and fungi we found in starters was high and not uniform. The number of microbial taxa (ASVs) in the starters was somewhat surprising, as it was much higher than previous estimates of global sourdough starter diversity (22). This result is due in part to our use of DNA sequencing-based approaches, which unlike traditional cultivation-based techniques, characterize both the bacteria and fungi that are easy to grow in the lab and those that are not. Despite our different method, we still found the expected dominance of yeasts and lactic acid-producing bacteria (23). Our reliance on DNA sequencing did not allow for plate counts or strain-level identification, but it had other advantages (24), including broader taxonomic coverage and greater power to characterize low-abundance samples (e.g., dust, hand swabs), thus enhancing comparability. Future studies might pair sequence-based approaches and plate counts or strain-level identifications on the same samples to elucidate how differences in approach alter our understanding of these communities.

The variation in starter composition we observed is of practical relevance in as much as it has the potential to influence the acidity of the starter as well as the flavor, texture, shelf stability, and nutrition of the bread produced using the starter. Indeed, we found that the pH of the starter and bread flavor were both associated with the microbial composition of the starter. It is known that the identity of bacteria in starters, especially lactic acid bacteria, can influence pH and other chemical properties of bread (20, 25). This association can occur because lactic acid bacteria produce different amounts of the particularly “acid”-tasting acetic acid (25) or through the production of esters, alcohols, and carbonyl compounds that can modify sourdough aromas (26). Our results corroborate this, as we found that starters in which bacteria of the *Lactobacillales* order were relatively more abundant were more acidic. In addition, we found that the composition of the bacteria in the starter, including specifically, the *Lactobacillales* component, influenced overall bread flavor. Future work could address whether specific members of the *Lactobacillales* are responsible for making more or different compounds which contribute to sourdough acidity and flavor.

A subset of the bacteria and yeasts found in starters was present in the flour used to make the starters but not on the hands of the bakers. These bacteria and yeasts were almost certainly from the flour itself, having colonized the flour either in the field or in milling, processing, storage, and transport. Indeed, more than half (∼60%) of the microbial taxa (ASVs) present in flour were also found in at least one starter sample. In other words, most microbial taxa found in flour successfully colonized at least some sourdough samples. However, this is not to say that the reverse was true, that most microbial taxa found in sourdough starters were uniquely from flour. A relatively large number of bacterial and yeast taxa (∼26% of all ASVs in starters) were shared between the skin microbiome on bakers’ hands and the sourdough starters but were not found in the flour. We suspect that these taxa colonized the starters from hands. We hypothesize that while the overall microbial community of starters was most similar to the flour used in their preparation (Fig. 5), bakers also introduced relevant species from their hands or bodies more generally during the making and processing of the starter. In addition, those taxa are more likely to be unique (not found in other sources) than are taxa from flour. This hypothesis is corroborated by the results of the SourceTracker analysis, which found hand samples to be the source of the greatest proportion of bacteria found in starters, although it is important to note that there are limitations to this approach and that it likely underestimates the role of unknown sources (27).

The idea that the microorganisms of mammals are key components of fermented foods is not novel. Of course, fermented dairy products include the microorganisms in the milk of the animal that produced the milk. They can also include the microorganisms of the skin of the animal that produced the milk. Cow skin bacteria, including strains of *Staphylococcus*, have been found in cheese (28). Other authors have commented on the potential of the bodies of bakers to contribute microorganisms to starters (5) or have found specific species of Lactobacillus plantarum and Lactobacillus spicheri on bakers’ hands that have also been found in sourdoughs (10). We appear to be the first to compare the skin microbiome of bakers’ hands to their starters in a systematic way. Admittedly, our analyses are limited by the fact that we do not have samples from the hands of bakers at the time when they initially prepared the sourdough starters or replicate samples of individual starters. Future research should include such paired samples as well as samples from nonbakers to more directly test if bakers have unique, food-influenced microbiomes. Additional studies could test if bakers and chefs more generally colonize the food they make as has been shown with the traditional production of chicha, a fermented cassava drink (29, 30).

It has been argued that each human has a unique microbial contribution that is a kind of fingerprint of his or her life (31); the same could, in theory, be true of each baker’s bread. We did find some evidence that the individual microbiology of each baker contributed to the uniqueness his or her starters’ microbiology, but the effect was weak. Based on our results, we could not predict the baker who made a starter based solely on his or her microorganisms. Why might we see many human microorganisms in starters but relatively little match between a particular baker and a particular starter? We can think of a few nonexclusive hypotheses. The first are technical. It may be that the unique microorganisms contributed by bakers to their starters are unique strains rather than higher taxa (ASVs) and thus were missed by our methodology. Amplicon sequencing identifies ASVs at a relatively coarse level, so approaches capturing more genes may be necessary to identify individual contributions to starters. Some of the relevant taxa may also have become too low in abundance either on human skin or in starters due to travel, washing, or other physical processes between the time of initiating the starters and when samples were collected. It is possible, for instance, that a microbe taxon is more common one day when a baker is working in a bakery (and has just touched another food or person) than it is the next day or week. Temporally rare species found on human or other animal bodies may have large effects in food fermentation. Our study design would miss such effects.

Other potential explanations are ecological. For instance, many of the yeasts and *Lactobacillales* bacteria found on skin could be relatively common among humans, or at least among bakers. We cannot rule out the possibility that the skin-associated microorganisms we found in the starters came not from the bakers themselves but instead from the air or equipment around the bakers (10), such that they may have come from the bakers but also other individuals with which they live and work. Finally, the process whereby microorganisms from hands colonize starters could be relatively stochastic. That stochasticity is a key element in the composition of the starter community is suggested by the observation that bacteria and fungi in starters were no less variable from one starter to the next than were the bacteria and fungi found on hands, even though the starters were all made using the same flour.

The relationship can also go the other way, with starters shaping the bakers working with them. While our study was not designed to test this explicitly, it does indicate the extent to which the skin microbiomes of bakers’ hands are unusual relative to the hands of nonbakers. In studies to date of the hands of students (17) and the general public (32, 33), the proportion of bacteria from the *Lactobacillales* has been relatively low. These bacteria tend to be more common on the hands of women than those of men, which has been thought to reflect incidental transfer of vaginal bacteria (human vaginal communities are dominated by *Lactobacillus* species) (17, 34). We expected to find something similar on the hands of bakers. Instead, we found that up to 27% (average, 15%) of the bacterial species and 88% (average, 33%) of the sequence reads on the hands of bakers were from species of the genus *Lactobacillus*. Our study also appears to show a much greater dominance of yeast on the hands of bakers than on hands sampled in other studies (33, 35). Though warranting more extensive research, we hypothesize that this dominance is due to the daily work of the bakers, which puts their hands in contact with dough, starters, and flours for many hours a day. It is possible that the skin microbiome of individuals’ hands more generally reflects the daily actions of their hands but that this effect has been missed because so many of the hand studies to date focus on students (who do not yet have a profession, especially one in which their hands are exposed repeatedly to a particular nonrandom group of microorganisms). Altogether, it seems that not only can bakers contribute microorganisms to their foods, but their daily work contributes microorganisms to them. In other words, the microorganisms on our hands may record not just who we are but also how we have lived, with bakers’ bodies specifically documenting their intimate relationships with bread.

## MATERIALS AND METHODS

### Experimental design.

The objectives of the study were to have bakers from different locations in Europe and North America make a spontaneously fermented sourdough starter using the same flour and recipe and then to compare the microorganisms of these starters to the potential sources of microorganisms, including bakers’ hands, flour, water, and dust. As a first step toward understanding the relationship between the starter microbial communities and bread acidity and flavor, each baker’s starter was used to make bread following the same recipe and using the same baking equipment. Dough acidity and bread flavor profiles were then assessed for each bread and compared with the starter microbial communities.

### Making the sourdough starter.

Professional bakers were recruited (*n* = 18) with the assistance of Puratos Company. Bakers were instructed to make starters in their home country using a standardized protocol (Fig. S1) and using flour that was shipped to them from a single source (stone-ground wheat flour; Epistar Tradition Francaise [T65-11/680]). Starters were made approximately 1 month prior to sampling. All sampling complied with the North Carolina State University institutional review board (NCSU IRB; IRB number 11952).

In short, on day 1 of the protocol, bakers mixed together a 1:1 mix of flour and unchlorinated water and maintained it at room temperature covered. On subsequent days, the sourdough starter was fed by removing 120 ml of starter then adding 240 ml of flour and 180 ml of water. Feeding was repeated daily until the starter doubled overnight. Subsequently, the starter was kept in the refrigerator and only fed once per week.

### Sampling microorganism sources.

**(i) Samples of unrefreshed sourdough starters, water, and dust.** Bakers traveled with their starters to a central baking location in Belgium for sampling and bread baking. Upon arrival on 4 July 2017, we sampled each baker’s unfed starter. “Unfed” or “unreplenished” refers to starters that had not been provided flour and water in the shared baking facility. Starters were maintained in various containers provided by the bakers for transport (most containers were glass). To sample the starter, a sterile swab (BD BBL) was pressed into the depth of the starter (without remixing the starter in the moment of sampling). After all starters were sampled, bakers were allowed to feed their starter with flour and water provided by the Puratos Co. (in the same room). At this time, we sampled the flour (stone-ground wheat flour; Epistar Tradition Francaise [T65-11/680]) that was sent to the bakers. To account for additional sources of microorganisms other than flour and hands, we sampled the facility’s tap water (as representative water) and dust from an interior facility door trim per the methods in reference 14. To the extent that the time spent in a common facility influenced starters (postfeeding), the expectation was that it would decrease variation in microbial composition.

**(ii) Samples of baker’s hands.** On the morning of 5 July 2017, as bakers arrived at the facility, but prior to handling their starter, their hands were swabbed (in the facility). Swabbing consisted of one dual-tip swab dipped in molecular-grade sterile water (MoBio) and then used to vigorously swab the front and back of both hands (but not in between digits). Swabbing took approximately 1 minute per person.

**(iii) Samples of refreshed sourdough starters.** A second sample of the now fed (or refreshed) starters was taken. This sample was taken with a sterile swab by brushing the surface of the unmixed starter. Additional control samples were taken of unused swabs and a swab with the sterile water used for hand sampling.

As expected, the process of feeding starters (adding additional flour and water) did not introduce large variation into the starter community. Starters were more similar to their prefed composition than they were to another random starter regardless of the distance metric taken into account (*P* ≤ 0.001, Mann-Whitney U test; Fig. S5). Of note, the high similarity of unfed and fed starters suggests little change in overall microbial composition due to (i) feeding, (ii) sampling depth (since unfed samples were sampled at depth and fed samples were sampled on their surface), and (iii) colonization of novel microbes over the short time periods involved in manipulation of starters. Because of the high similarity between refreshed and unrefreshed starters (Fig. S5) and in order to avoid pseudoreplication, we only included unrefreshed starter samples in our analyses. Due to a temporary loss of luggage, one baker did not have a sample of fed sourdough starter.

10.1128/mSphere.00950-19.5 FIG S5 Correlation between unfed (unrefreshed) and fed (refreshed) sourdough starter bacterial (A, B) and fungal (C, D) communities. Comparisons between refreshed and unrefreshed samples from the same starter (purple) have much lower dissimilarity than comparisons between refreshed and unrefreshed samples from different starters (green). Boxplot shows the median and the first and third quartiles, and whiskers show 1.5 times the interquartile range. *, *P* < 0.001 Mann-Whitney U test. Download FIG S5, TIF file, 0.4 MB. Copyright © 2020 Reese et al. 2020 Reese et al. This content is distributed under the terms of the Creative Commons Attribution 4.0 International license.

### Starter chemical analysis.

The pH of each refreshed starter was measured prior to baking using a pH meter (Knick; Elscolab). Total titratable acidity (TTA) was determined by titration (Titrino 848; Metrohm). Briefly, 10 g of sourdough (prior to baking) was homogenized with 100 ml of distilled water and neutralized with NaOH 0.1 N. The acidity is expressed as the volume in ml of NaOH 0.1 N added to read a pH of 8.4. Starter pH and TTA were highly correlated (*P* < 0.001, rho = –0.92, Spearman correlation; Table S1), so only analyses of pH and microbial composition are reported. All trends found for pH and microbial composition were also found for TTA.

### Bread baking and sensory analysis.

Following sampling of the refreshed starters, bakers proceeded to make a bread using shared equipment, shared space, and a standard recipe. Each baker used his or her own starter to make a bread following a standardized recipe, ingredients, methods, and equipment (Fig. S2). In order to screen the high number of breads (*n* = 17, with an additional sample used as the reference) for flavor attributes, sensory free sorting was performed. An untrained panel of judges (the bakers) was presented with all the breads and asked to provide five descriptors of that bread, along with an intensity score.

Following the identification of the five dominant descriptors of all the breads, a panel of expert judges (different from the bakers) scored each bread for flavor intensity from 0 to 10 for each of those five descriptors (Table S5). One of the breads was chosen as a reference bread according to its average values for descriptors. Each bread was scored by 8 to 9 judges. Judging took place over three sessions in order to limit the number of breads assessed by any one judge.

### Molecular analysis.

**(i) DNA extraction.** The swabs were all kept at –20°C following collection. Swabs were transported on ice to the Laboratory of Molecular Bacteriology at the Department of Microbiology and Immunology of KU Leuven (Belgium), where DNA was extracted using the DNeasy PowerSoil kit (Qiagen, formerly MoBio) with small modifications. In line with Fierer et al. (17), an extra heating step was added after the addition of the lysis solution C1 at the start of the protocol. At the end of the protocol, 5 minutes of incubation time was added to allow resuspension of the DNA on the filter before elution (step 19). The remaining steps were performed per the manufacturer’s protocol. Extracted DNA was mixed with DNAstable plus (Biomatrica) per the manufacturer’s protocol and shipped to the United States for further amplification and sequencing.

**(ii) DNA amplification and sequencing.** Microbial communities were characterized following the molecular and bioinformatics protocols described by Barberan et al. and Oliverio et al. (14, 36). Briefly, this included amplifying the v4 region of the 16S and the ITS region of ribosomal DNA with barcoded primers to allow for sequencing in multiplex. PCRs were performed for each of the 60 extracted DNA samples, and we included and sequenced multiple negative “no template” controls to check for possible reagent contamination. Samples were normalized and cleaned with Sequalprep normalization plates (Invitrogen) prior to sequencing in multiplex on the Illumina MiSeq platform at the University of Colorado BioFrontiers Next Generation Sequencing Facility with the 2 × 250-bp paired-end chemistry for a mixed ITS/16S run.

**(iii) DNA sequence processing.** Raw sequences were demultiplexed and processed as exact sequence variants (ASVs) with the DADA2 pipeline (37), as described at https://github.com/amoliverio/dada2_fiererlab. The DADA2 pipeline resolves sequence variants rather than clustering by a percent identity threshold. For both ITS and 16S, reverse reads were discarded due to poor quality. We quality filtered reads for both to a maximum expected error threshold of 1. For the ITS data, we set truncQ = 2 and minLen = 150, and for the 16S data we set truncQ = 11 and truncLen = 240. Then we inferred sequence variants with the DADA2 algorithm (37), constructed a sequence table, and removed chimeras. Taxonomy was assigned using the Silva database version 132 (38) for 16S reads and with the UNITE database version 2.02 (39). 16S reads assigned to chloroplasts, mitochondria, or *Eukaryota* were removed prior to downstream analyses. For both 16S and ITS data sets, reads not assigned a taxonomy at the phylum level were also removed.

Samples resulted in 2,068 to 10,341 bacterial sequences for hands, 3,617 to 9,135 bacterial sequences for starters, 1,666 to 29,293 fungal sequences for hands, and 1,579 to 30,705 fungal sequences for starters. All raw data are publicly available through the European Nucleotide Archive under accession number PRJEB34686.

Analyses including only hands and unrefreshed starter communities were carried out on a rarefied data set, where 16S sequences were subsampled to a constant sequencing depth of 3,500 reads, and ITS sequences were subsampled to 750 reads. Rarefaction removed environmental samples, however, so analyses including flour or other environmental samples were conducted on the unrarefied data set.

### Statistical analysis.

All analyses were carried out in R version 3.3.3. Alpha- and beta-diversity metrics were calculated with the package vegan (40). Alpha-diversity was calculated as ASV richness and Shannon index. These values were compared for the bacteria or fungi and the *Saccharomycetales* and *Lactobacillales* using Mann-Whitney U tests to compare hand swab to starter samples. The effect of baker identity was tested with Kruskal-Wallis tests. The association between pH and TTA was tested with Spearman correlation.

Beta-diversity between samples was calculated either as abundance-weighted Bray-Curtis dissimilarity or presence/absence Jaccard index using the ASV tables for hand, unrefreshed starter, flour, and water samples or unrefreshed and refreshed starter samples. Permutational multivariate analysis of variance (PERMANOVA) was conducted using the adonis function in the package vegan to test for relationships between sample type, baker identity, and starter acidity on the composition of microbial communities. Nonmetric multidimensional scaling (NMDS) plots were produced with Bray-Curtis distance calculations using the metaMDS function.

To compare a baker’s starter with his or her hands relative to other hands, we calculated Bray-Curtis or Jaccard beta-diversity measures among all hand and starter samples. We included only taxa present in at least one starter and one set of hands to enhance the potential overlap between communities. We then extracted the distances for a baker’s starter versus hand sample as well as for a baker’s starter versus a random hand sample (selected with a random number generator). These were then compared with the Mann-Whitney U test.

SourceTracker (18) was applied using default settings to the combined 16S and ITS data sets including all environmental (water, dust, and flour), hand, and starter samples to assess which served as likely sources for microbial colonization. First, we ran a model where the environmental and hand samples were potential sources for the sourdough starter communities. Then we ran a model where the environmental and starter samples were potential sources for hand swab microbial communities. SourceTracker is often used to statistically identify the origin of microbes in mixtures derived from multiple sources, such as in water supplies (41). Our use of this analysis is in line with this common application.

In the flavor analysis, we took the average of all scores (*n* = 8 to 9 except for one reference bread, which had *n* = 1) to estimate each flavor. Most bakers judged the bread flavors similarly (average standard deviation was 1.9). We compared overall flavors to overall composition with Mantel tests (using the mantel function in vegan) and individual flavors to microbial composition with PERMANOVA.

### Data availability.

The DNA sequences referenced in the manuscript are available in the European Nucleotide Archive under accession number PRJEB34686.
