# Supplementary figures and images for: Influences of Ingredients and Bakers on the Bacteria and Fungi in Sourdough Starters and Bread
Source: mSphere. 2020 Jan 15;5(1):e00950-19. doi: 10.1128/mSphere.00950-19 (PMC6968659; doi:10.1128/mSphere.00950-19)

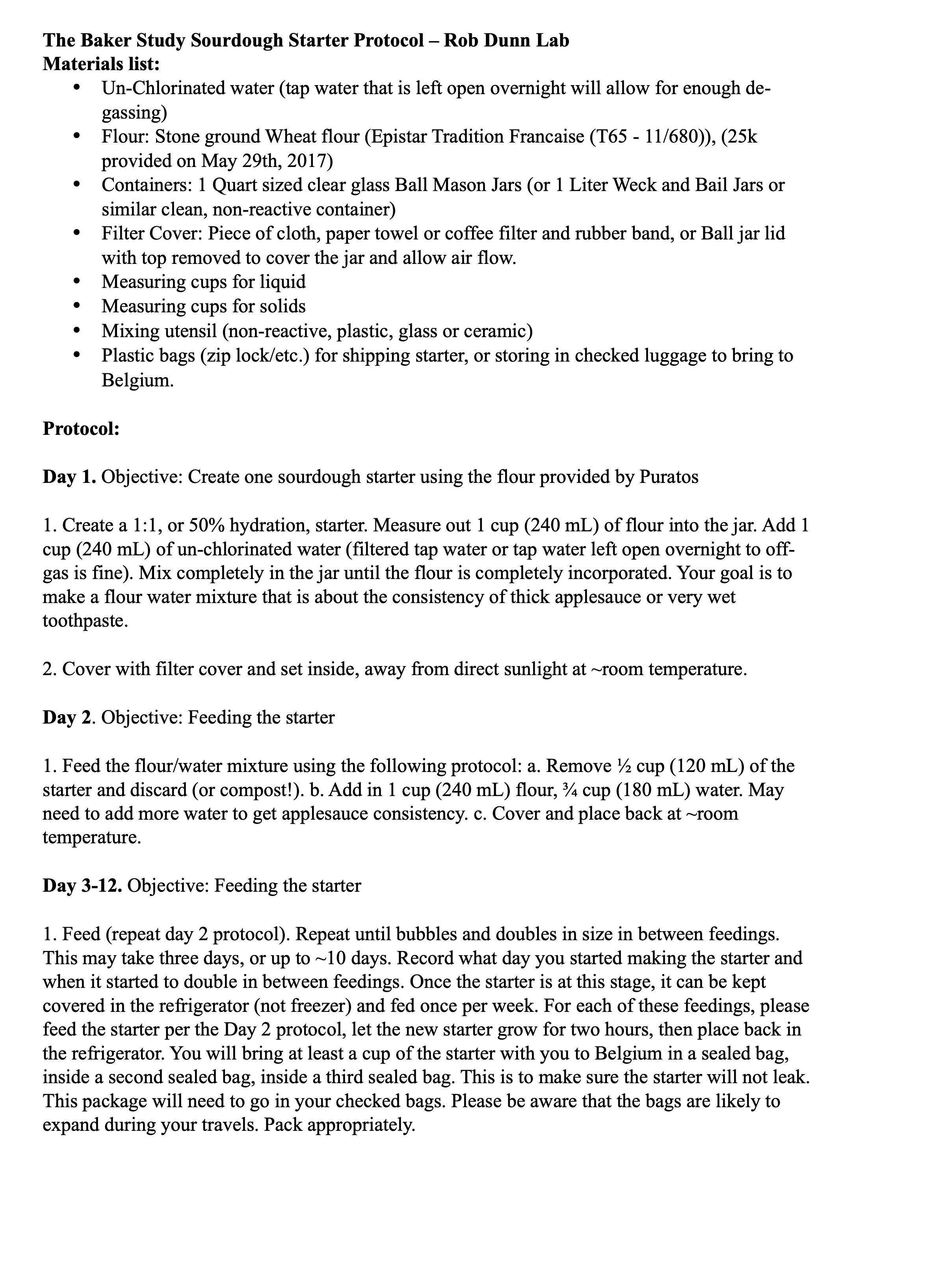

Supplement: Fig. S1 [file mSphere.00950-19-sf001.tif]

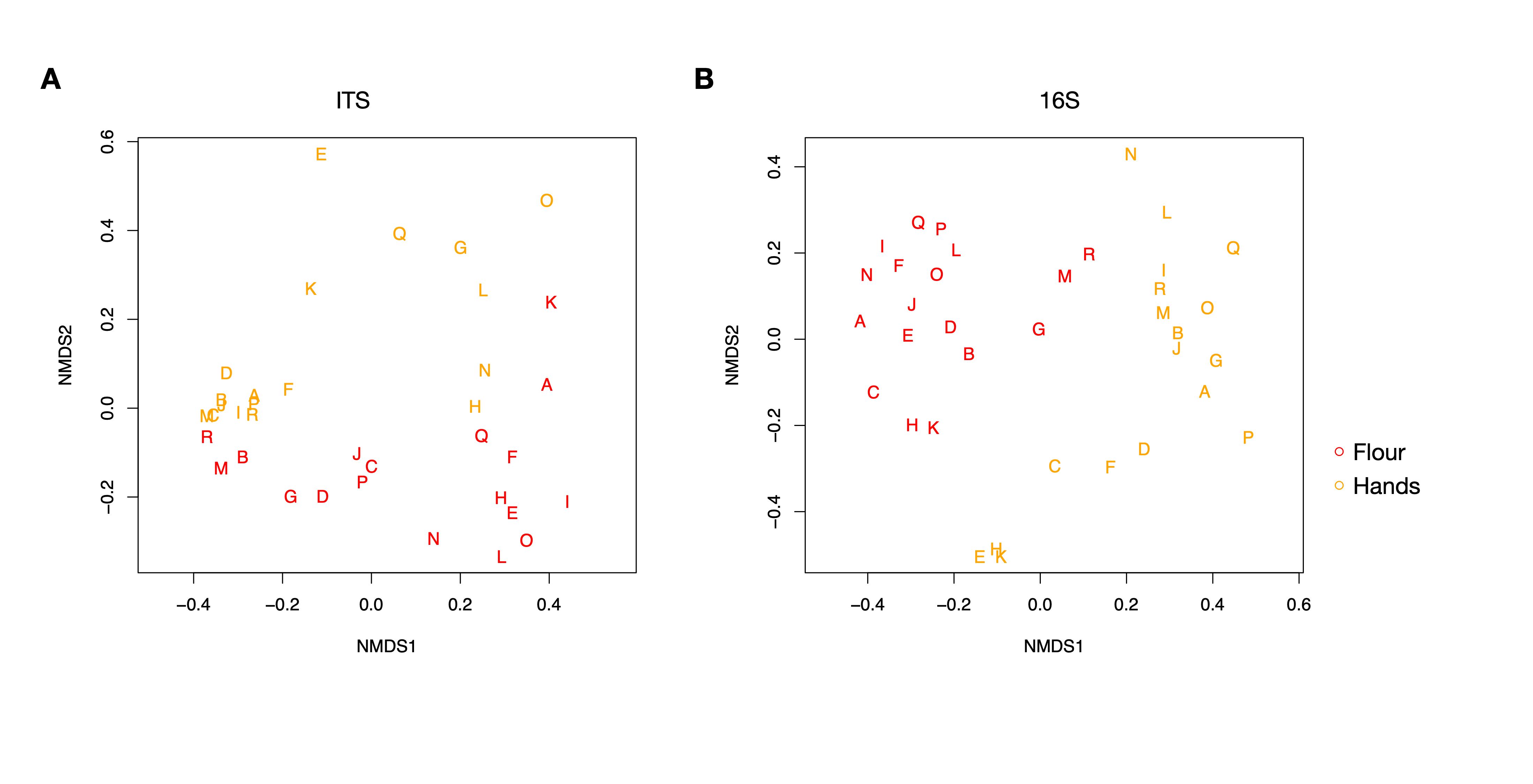

Supplement: FIG S3 [file mSphere.00950-19-sf003.tif]

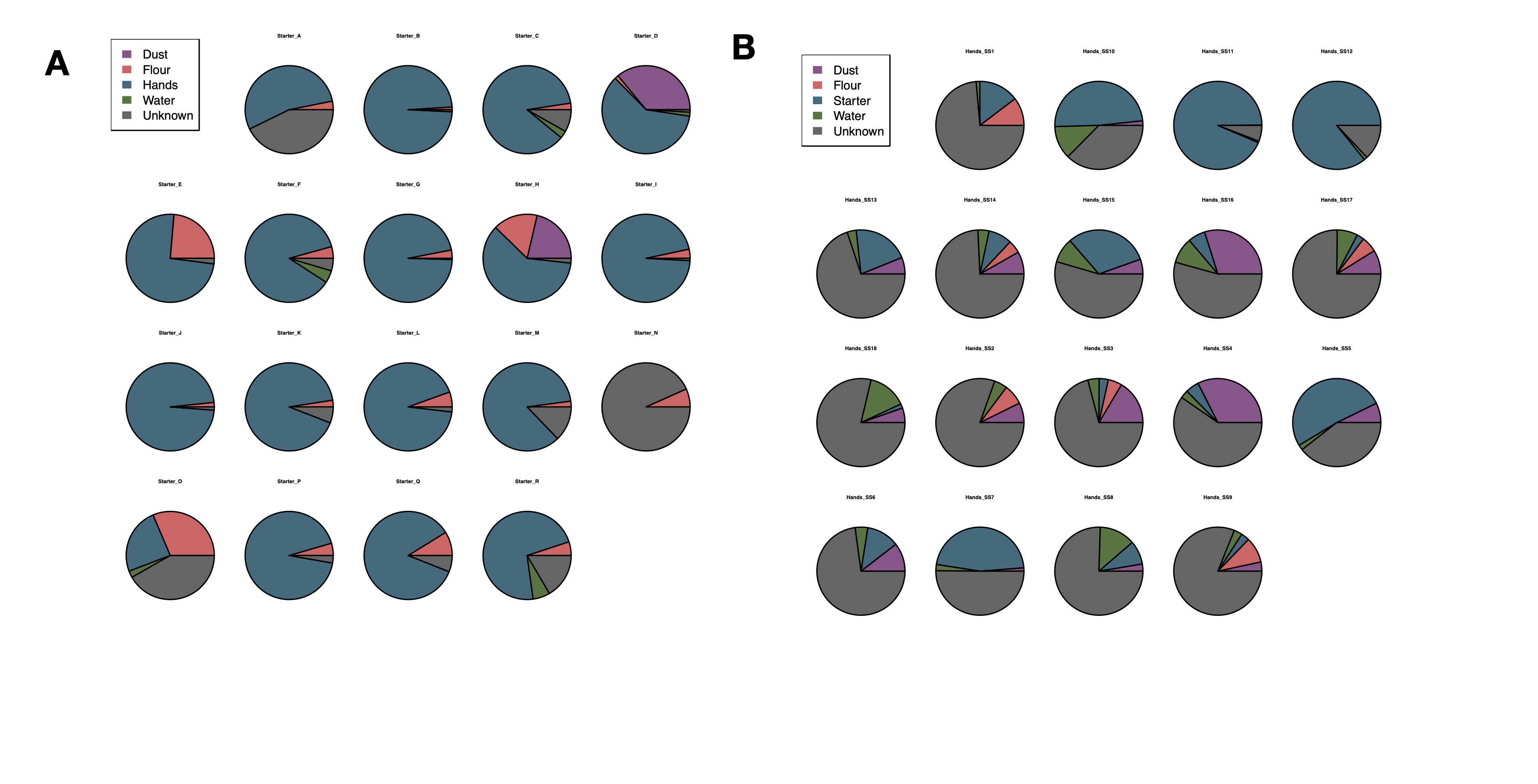

Supplement: FIG S4 [file mSphere.00950-19-sf004.tif]

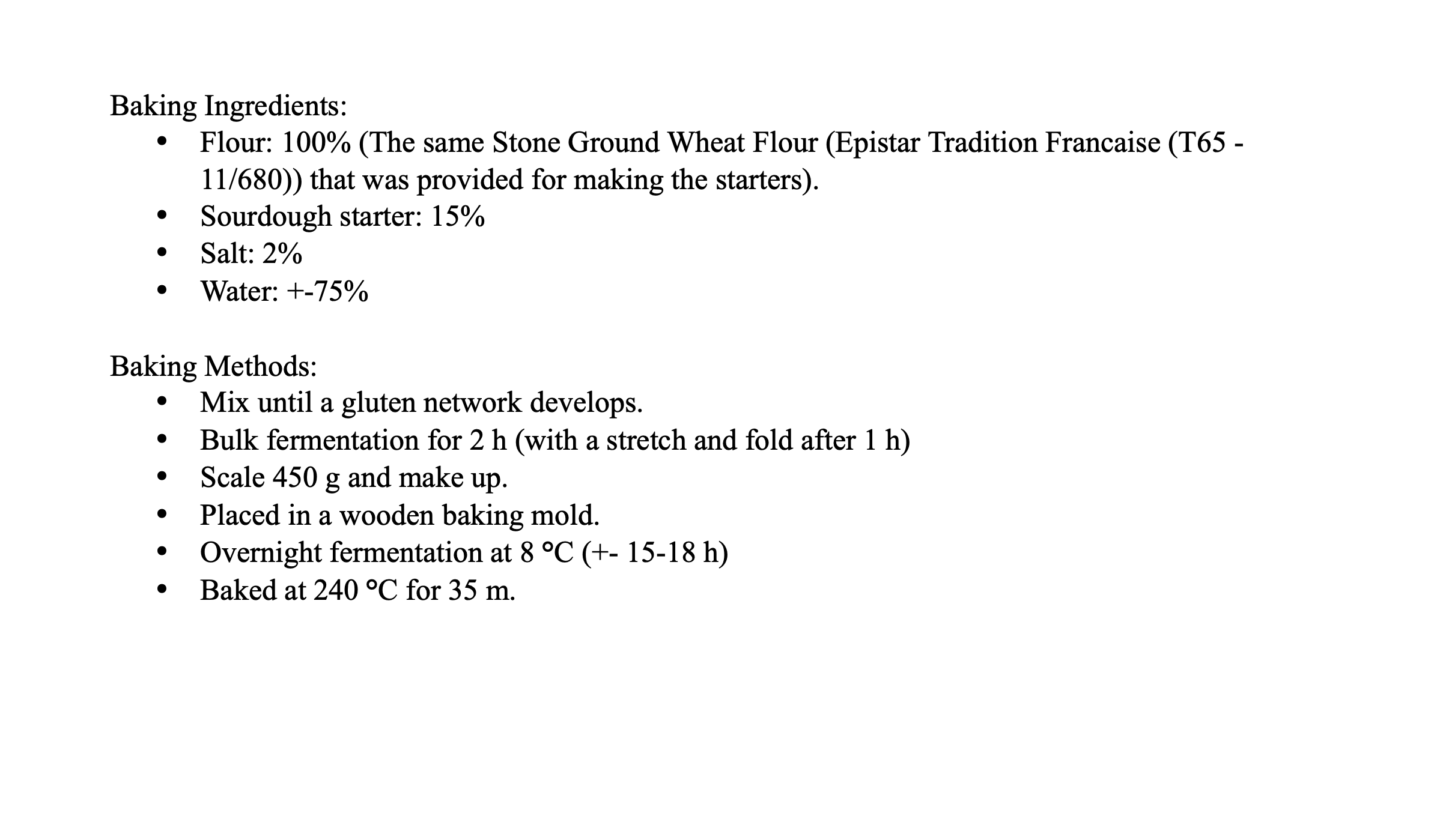

Supplement: FIG S2 [file mSphere.00950-19-sf002.tif]

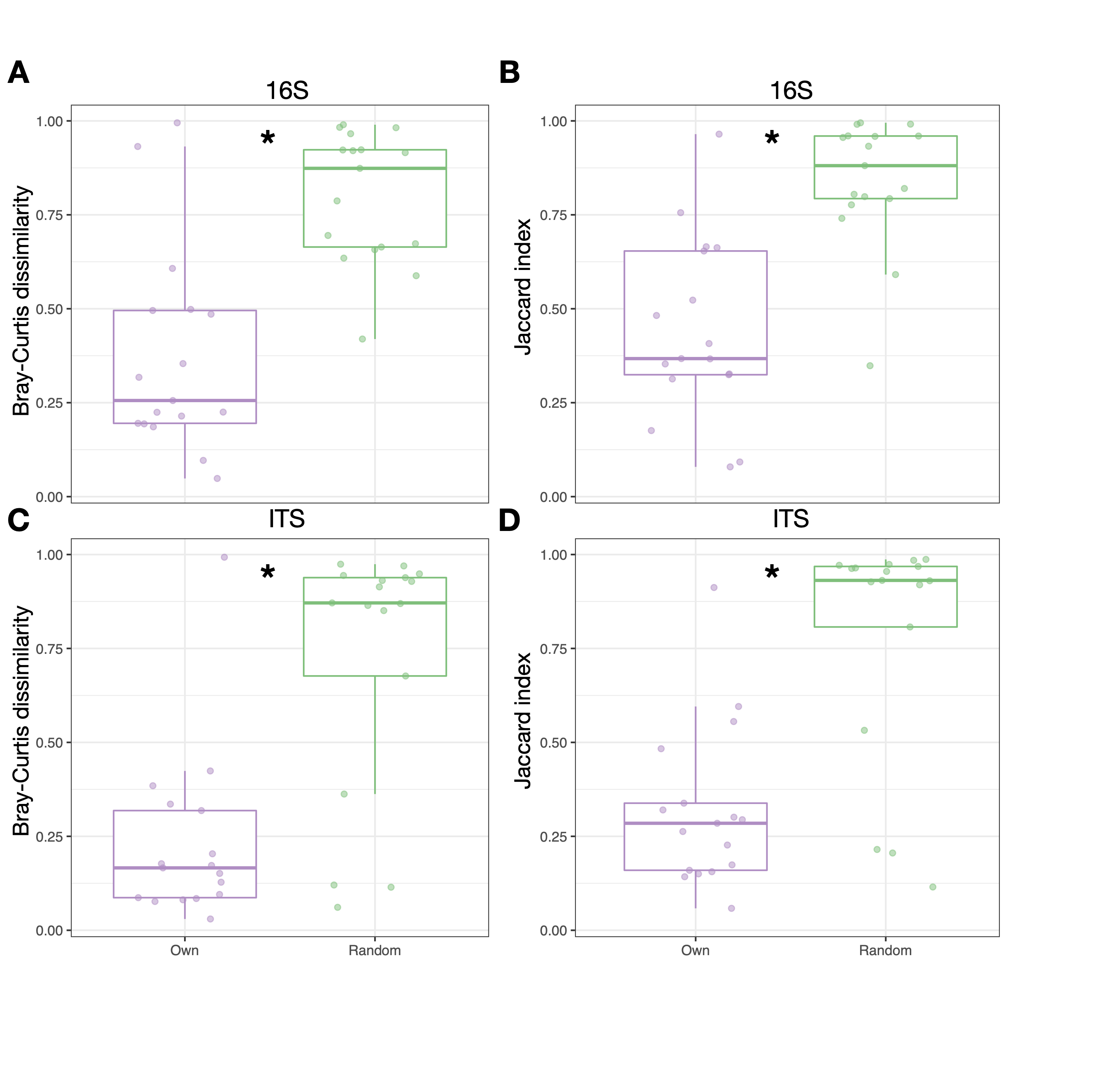

Supplement: FIG S5 [file mSphere.00950-19-sf005.tif]
